# Blurred lines: Ethical challenges related to autonomy in home-based care

**DOI:** 10.1177/09697330231215951

**Published:** 2023-12-20

**Authors:** Cecilie Knagenhjelm Hertzberg, Anne Kari Tolo Heggestad, Morten Magelssen

**Affiliations:** 6305 University of Oslo; 6305 University of Oslo; 6305 University of Oslo

**Keywords:** Autonomy, clinical ethics, coercion, home-based care, participant observation, qualitative research

## Abstract

**Background:**

Home-based care workers mainly work alone in the patient’s home. They encounter a diverse patient population with complex health issues. This inevitably leads to several ethical challenges.

**Aim:**

The aim is to gain insight into ethical challenges related to patient autonomy in home-based care and how home-based care staff handle such challenges.

**Research design:**

The study is based on a 9-month fieldwork, including participant observation and interviews in home-based care. Data were analysed with a thematic analysis approach.

**Participants and research context:**

The study took place within home-based care in three municipalities in Eastern Norway, with six staff members as key informants.

**Ethical considerations:**

The Norwegian Agency for Shared Services in Education and Research evaluated the study. All participants were competent to consent and signed an informed consent form.

**Findings:**

A main challenge was that staff found it difficult to respect the patient’s autonomy while at the same time practicing appropriate care. We found two main themes: Autonomy and risk in tension; and strategies to balance autonomy and risk. These were explicated in four sub-themes: Refusing and resisting care; when choosing to live at home becomes risky; sweet-talking and coaxing; and building trust over time. Staff’s threshold for considering the use of coercion appeared to be high.

**Conclusions:**

Arguably, home-based care staff need improved knowledge of coercion and the legislation regulating it. There is also a need for arenas for ethics reflection and building of competence in balancing ethical values in recurrent ethical problems.

## Introduction

Health care is not value-neutral; it is an activity that promotes human life and health through healing, palliating, comforting, and preventing. The health worker–patient relationship is asymmetric; with professionals’ power comes moral responsibility.^1^ This may lead to value conflicts and, thus, ethical challenges. Home-based care differs from other care settings as care is provided in the patient’s home.^2^ Care is given regularly, usually daily. The patient’s home is typically not adapted for care, and the patient may live with a partner or children. Staff primarily work alone as ‘visitors’ in the patient’s home,^3^ and the patient’s health situation is often complex and varied, typically involving multiple somatic and/or mental illnesses.

Today patients are discharged from hospitals earlier than before, and the number of patients who receive treatment and care at home has increased. Generally, patients cared for at home are more vulnerable and sicker than before.^4–7^ This may lead to more complex ethical challenges.

The project as a whole aimed to explore the ethical challenges experienced by staff in home-based care and what characterizes these challenges, and how patients and relatives experience receiving care at home. In this article, we aim to explore and discuss ethical challenges related to patient autonomy in home-based care and how staff deal with such challenges. We discuss the findings in light of Beauchamp and Childress’ principle-based ethics.^8^

### Definition of key concepts

The three main concepts in this article are ethical challenge, autonomy, and coercion.

We can define an *ethical challenge* as when norms or values collide and there is doubt, or disagreement about what is right or wrong.^1^ An ethical challenge can also be defined as a situation where there is a conflict between moral principles.^8^

Honouring *the principle of autonomy* in healthcare means that the patient’s self-determination is recognised, that is, that the patient’s opinions and decisions are acknowledged and that staff act on the basis of the patient’s personal values and beliefs.^8^

Health professionals sometimes find that they are unable to act in accordance with the patient’s autonomy. Actions might sometimes amount to *coercion*, which can be defined as when someone intentionally uses a credible and serious threat of harm or force to manipulate or control another person.^8^

### Previous research on ethical challenges related to autonomy and coercion in home-based care

Several studies on home-based care report ethical challenges related to autonomy and self-determination.^2,9–22^ Patient autonomy is particularly difficult when the patient is cognitively impaired.^23–26^ Employees in home-based care face the ethical challenge of balancing patient autonomy with principles of beneficence, non-maleficence, and justice and often have difficulty assessing the patient’s capacity to consent. In addition, staff face ethical dilemmas when patients do not want to follow staff recommendations.^27–30^ According to a Dutch study,^28^ patients living at home have specific wishes regarding how they want to be treated, and it is essential that staff at home-based care respect this.^28^ Other studies show that mutual trust and respect are crucial for maintaining a relationship.^14,22,31–38^

There is a lack of knowledge about how home-based care workers manage patient autonomy. Additionally, most studies on the topic focus on care for patients suffering from dementia. The present study includes patients with and without dementia and provides detailed insight into the actual situations that involve ethical challenges related to patient autonomy and how these are handled by staff.

## Method

The study is based on participant observation and interviews in three municipalities in the Eastern part of Norway from September 2020 to November 2021.

Participant observation provides a unique opportunity to delve into the intricacies of human behaviour.^39,40^ In this method, one must immerse oneself in the lives of the research participants in order to gain a deeper understanding of the field by observing uncontrolled, real-life situations.^39^ The interviews, on the other hand, allow individuals to articulate their personal experiences and address questions that arose during the participant observation and validate the observations.^39^ The combination of participant observation and interviews gives a rich data material.

We also know that it may be difficult to put ethical challenges into words^41^; thus, participant observation can capture and shed light on these challenges in the daily work of the participants.

### Context: the three municipalities

Municipality 1 was a small, rural municipality with a population of about 18 000. Patients were spread over a large area, lived in detached houses, and many had no neighbors nearby.

Municipality 2 was a middle-sized municipality with a population of about 35 000. Home-based care was divided into three units: two somatic units and one dementia and geriatric psychiatry unit. The data collection took place in the latter unit. The area was rural and urban, and patients lived in detached houses or apartment buildings.

Municipality 3 was a large city divided into districts. Home-based care in the relevant district was divided into three areas: X, Y, and Z. Data collection took place in areas X and Y. In area X, the houses were well-equipped, and the apartment buildings were new and modern. In area Y, the accommodations were more diverse. Some lived in public housing or small, cramped old flats, mostly without elevators.

### The participants

Two staff members in each of the municipalities were recruited by their managers as key informants (Table 1). In this article, we use the terms staff or workers as common terms for the employees (nurses, auxiliary nurse, health workers, and unskilled workers) in home-based care. Names used in this article are fictional.

**Table 1.** Overview of key informants.

Municipality	Pseudonym	Profession	Working years
1	Anne	Palliative nurse	6 to 10 years
	Line	Auxiliary nurse	16 to 20 years
2	Emilia	Nurse	1 to 5 years
	Silje	Psychiatric auxiliary nurse	6 to 10 years
3	Elise	Nurse	1 to 5 years
	Ida	Nurse	16 to 20 years

The staff recruited 45 patients based on patients’ ability to consent, health status, and desire to participate (Table 2

**Table 2.** Overview of patients.

Municipality	Patients recruited	Main health issues
1	15	Cancer; multiple sclerosis; dementia; chronic obstructive lung disease; heart and lung disease; mental illness; substance abuse; liver failure; diabetes; old age
2	8	
3	22	

).

### Participant observation

During the participant observation (Table 3

**Table 3.** Fieldwork.

Municipality	Time	Number of days conducting participant observation
1	September–November 2020	8 (56 h)
2	March–May 2021	15 (92 h)
3	August–November 2021	18 (122 h)

) the first author (CKH) assisted in caring for the patients (e.g. hygiene, cleanliness, and food). Because of restrictions due to the pandemic, she only got to observe for 8 days in municipality 1. In municipalities 2 and 3, COVID-19 still influenced daily life, but at this stage there were fewer restrictions.

Observations and conversations were written as fieldnotes on an iPad. These amount to 95 pages.

### Interviews

In municipality 1, CKH and AKTH conducted a focus group interview with key informants and five home-based care workers (Table 4

**Table 4.** Overview of informants in focus group interview (municipality 1).

Pseudonym	Profession	Working years
Marie	Nurse	20 to 25 years
Mathilda	Nurse	10 to 14 years
Andrea	Nurse	1 to 5 years
Helga	Nurse	16 to 20 years
Sonja	Auxiliary nurse	16 to 20 years
Anne (key informant)	Palliative nurse	6 to 10 years
Line (key informant)	Auxiliary nurse	16 to 20 years

). The original plan, which had to be changed due to COVID-19, was to have focus group interviews in all three municipalities. COVID-19 brought restrictions on the number of people who could be in a room. We therefore decided to conduct separate in-depth interviews with the key informants in municipalities 2 and 3, one joint with Emilia and Silje and another joint with Ida and Elise, thus two interviews with four key informants in total.

The first author used a semi-structured interview guide with follow-up questions. The themes of the interview guide were as follows: (A) Working in home care; (B) ethical challenges or/and difficult situations; (C) communication and coordination; (D) caring for patients; (E) ethics support. All interviews were recorded, lasted between 1 and 2 h, and were transcribed by the first author. The total amount of pages was 95.

### Analysis

In ethnographic fieldwork, the analysis process begins when the researcher enters the field.^39^ We analysed the fieldnotes and the transcribed interviews in line with Braun and Clarke’s reflective thematic analysis. The first author did the main analysis. All three authors met several times to identify discuss, develop, review, and refine patterns and themes. We moved back and forth between the six phases described by Braun and Clarke.^42^ (1) Familiarizing ourselves with the data: CKH recognised patterns throughout the fieldwork. These patterns were further confirmed and discussed as the authors read the transcribed interviews and fieldnotes. (2) Generating initial codes: CKH began initial coding or categorizing of the data material, as she read through the fieldnotes and transcribed the interviews several times. (3) Searching for themes: The authors met to discuss preliminary themes that covered the coding and CKH systematised them in matrices. (4) Reviewing themes: The authors checked whether the themes worked in terms of the codes and the data set. (5) Defining and naming the themes: In this phase, the three authors defined and refined themes and subthemes. (6) Producing the report.

The analysis was thus an ongoing and reflexive process, from the initial phase of the fieldwork, through the interviews and transcribing, to the continuous reading of the data material.

### Ethical considerations

This project was assessed by the Norwegian Agency for Shared Services in Education and Research (project number: 980490). In addition, the project was exempted from the duty of confidentiality by the regional committee for medical and health research ethics (REC South East Norway project number: 130005).

The key informants and the patients signed a written consent form before the fieldwork. Other staff signed during data collection. All participants were informed that they could withdraw from the project at any time.

The first author and the participants did not know each other before the data collection. It is important to act cautiously when conducting participant observation of vulnerable groups, in this case patients, as many are unable to preserve their own interests.^18^ The first author considered her role in all settings with patients. It was important to her that they felt comfortable in her presence. She assisted in the care of patients, many of them in vulnerable situations, such as patients receiving palliative care or patients with dementia (but still competent to consent). Before entering a patient’s home, she always discussed her role with the key informant.

## Findings

The results are presented as two main themes and four subthemes (Table 5).

**Table 5.** Themes and subthemes.

Themes	Subthemes
1. Autonomy and risk in tension	1. Refusing and resisting care
	2. When choosing to live at home becomes risky
2. Strategies to balance autonomy and risk	1. Sweet-talking and coaxing
	2. Building trust over time

The first main theme represents the ethical challenges related to autonomy, while the second theme represents the staff’s strategies when handling these ethical challenges.

### Autonomy and risk in tension

All informants found it difficult to respect the patient’s autonomy while practicing appropriate care that did not put the patient at risk. This was a recurring ethical challenge, involving the delicate balance between patient autonomy and the principles of non-maleficence and beneficence.

#### Refusing and resisting care

Patients refusing or resisting care was the most common ethical challenge. This created difficulties for staff because they had to balance respect for patient autonomy and the practice of beneficial and non-maleficent care. The informants were thus in a professional but also personal dilemma, wondering what the right thing was to do: to let the patient’s autonomy override their own values as health workers, or the opposite, to override the patient’s autonomy and follow their own need to practice beneficial and non-maleficent care. We identified two patterns in the data related to patient refusal or resistance of care: high or low risk when refusing or resisting care.

High risk from refusing or resisting care occurred when the patient’s condition was critical or unclear and there was a risk to life and health. In these cases, informants had to make the decision whether to prioritize the patient’s life and health and thus override autonomy or to respect the patient’s autonomy yet accept the risk for the patient.

In the interviews and conversations, all informants expressed that they prioritised practicing beneficent care when a patient refused vital help. For example, Ida in municipality 3 explained the following in an interview:

There are various ways of resisting help. It can be acute when you see the patient getting sicker and sicker, pale and clammy and breathing fast. But the patient does not want you to call the emergency unit; he does not want anything, so I assess whether life and health are at stake. Because if I think life and health are at stake, I have to call, even if the patient is lucid and oriented. So, then I have to overrule him.

Informants said it felt terrible to overrule a patient’s autonomy in their home. They describe the fear of violating the patient’s trust, especially since they typically had an ongoing relationship with the patient.

Low risk of refusing or resisting care meant that the situation did not pose an immediate threat to life and health, such as when the patient refused help with hygiene or nutrition. In these cases, the informants typically adopted a ‘wait-and-see’ attitude and decided to act only when absolutely necessary, even if they did not agree with the patient’s decision. If the situation got out of hand, informants had to weigh what carried the most: the patient’s autonomy or beneficial and non-maleficent care.

In these situations, the imperative to overrule the patient’s autonomy and provide care was not as strong, so most commonly the decision entailed respecting the patient’s wishes. However, staff explained that it was challenging when patients’ refusals of care would become more critical in the long run, for example, if patients refused food, medication, or help with hygiene for an extended period.

Several informants stated that patients refusing to eat was common in home-based care. It was particularly difficult when the patient had dementia, as staff could not be sure whether a forgetful patient was telling the truth or not. In these cases, the staff had the choice to trust or distrust the patient and thus to respect the patient’s autonomy or not. During the focus group interview in municipality 1, Marie said:

They forget to eat, and then there is [the actual] eating situation; it is difficult for us to follow up when they say, ‘no, I am not hungry’.

Furthermore, informants claimed that many patients do not understand the importance of basic hygiene and often rejected this type of care and thus ran the danger of complications such as infections or septicemia. This is in line with CKH's observations; she saw that patients often refused help with diaper changes, catheter care, or diabetic feet and the staff respected this even though it could potentially become a genuine risk to health.

#### When choosing to live at home becomes risky

Many patients refused to move into a nursing home even though living in their home could pose a threat and risk to their life and health.

The risk of living in their home was often related to their health conditions, cognitive impairment, and poor judgment or to practicalities and hygiene within their residences.

The informants struggled to help the patient understand the potential danger, especially when longstanding habits were involved. In these cases, the informants had to respect patient autonomy, thus overlook the patients’ old habits and routines, but at the same time ensure that they were safe and healthy. During the focus group interview in municipality 1, Mathilda gave an example of a typical patient’s habit of lighting a fire in the wood stove:

It is not easy, because you cannot change when older people have been lighting a fire in the wood stove for all these years. So, it's not easy to say you cannot because they may not remember it either. It´s part of their morning routine. There are many dilemmas like this.

The case described by Mathilda shows one of the dangers of living at home.

Several patients were living in circumstances which made it difficult to provide medical care in the patient’s home. Staff had to work with what was available to them and adapt their care to the patients’ lifestyle and preferences. From the observations and conversations, it was clear that the staff accepted working under these conditions because they valued the autonomy of the patients.

An example that illustrates the circumstances in which staff work and how much they respect patient autonomy is the case of Elliott. CKH visited Elliot, a patient in municipality 3, several times. Below is a description of the first meeting with him:

We often passed his window, but it was dark. Tiny dolls dangle by threads in the windows; they look as if they are being hanged. Ida tells me to wear a face mask because of the smell and not to touch things unnecessarily. The entrance door, which used to be white, is dirty with fingerprints. Ida locks us in and says good morning. It is dark and the smell of urine hits me. We go into the kitchen; there is an elderly man lying in an old wooden bed; the bedding is grey and brown from dirt... Ida turns on the light and Elliot quickly jumps out of bed. He is wearing a red-stained shirt and big, dirty jeans (...). The kitchen is worn and dirty and in disarray. There are several buckets filled with water and clothes on the floor. Elliot does not want home-based services to clean, tidy or keep the flat in a better condition (fieldnotes, municipality 3).

Elliott had no hot water, no bath, and no toilet in his flat. Ida, the key informant, explained that Elliot’s situation had reached a point where it became difficult to provide proper appropriate medical assistance. They struggled to maintain cleanliness and hygiene when attending to some of his wounds. Preparing food was difficult due to the untidy and dirty kitchen. Ida mentioned during the fieldwork that they (home-based care) had given Elliot an ultimatum: either he accepted help with housekeeping, or they would no longer provide care. The commitment of an appropriate care was then judged to be more important than Elliot’s self-determination.

In many situations the patient was additionally cognitively impaired, which made the situation at home even more dangerous. During one of our car trips, key informant Line from municipality 1 told CKH about a patient with severe dementia who lived alone in a house in the forest. His house was unsuitable, he slept on the kitchen ground, had a fireplace right on the wooden floor, and used a gas cooker. The staff in municipality 1 felt that it was dangerous for him to live there. Line and CKH discussed this issue in the car:

We talk about how advanced the dementia diagnosis is in some patients. Most [of the staff] agree that many of the patients Line and I visited today should not be living alone at home. But it's not easy to admit someone. For lack of space, but mostly because the patient does not want to [move to a nursing home or assisted living facility].

### Strategies to balance autonomy and risk

As a way to balance the patient’s autonomy and practice beneficent and non-maleficent care, we discovered that informants used two main strategies. Firstly, what we understand as ‘sweet-talking and coaxing’ and secondly, building trust over time. We additionally saw that staff did what they could to avoid coercion but also that they had little knowledge about their legal right to write a formal decision on coercion.

#### Sweet-talking and coaxing

The informants had developed a strategy to handle patients who refuse or resist care. This strategy enabled them to respect the patient’s autonomy while being able to provide beneficial care. Staff would typically describe their strategies with the Norwegian colloquialism ‘lirke og lure’, which we translate as ‘sweet-talking and coaxing’. By ‘sweet-talking and coaxing’, we mean persuasive communication or behaviour intended to influence the patient to accept care. ‘Sweet-talking and coaxing’ did not designate a precisely delineated set of practices but rather encompassed a broad range of measures to get the patient to accept care. The informants could sweet-talk and coax over a long period of time before the patient accept care. As described in the fieldnotes from municipality 3 described:

Ida explains that much of the work is getting the patient to do things, sweet-talking and coaxing. This applies to everything and especially in this area where many live alone (fieldnotes, municipality 3).

According to informants, sweet-talking and coaxing could also be characterized as a kind of encouragement to gently push the patient towards the decision or behaviour desired by staff. Sometimes informants distracted the patient by, for example, leading them to the bathroom. Humour and kindness were also important factors in the strategy. This was especially evident in situations such as hygiene and nutritional measures, refusal of medication and refusal of help with housekeeping.

The informants could also entice the patient with some kind of reward, as Mathilda in municipality 1 told:

One patient I visit, I shower her once a week. It took a really long time for her to want to shower, but then I understood that she liked her hair rolled up, and that was a start [to get her to shower], now I fix her hair every time (focus group interview, municipality 1).

In the case described above, we can see that Mathilda enticed the patient by sweet-talking and coaxing. The result was that the patient showered regularly while still maintaining her autonomy.

CKH observed that there are some more questionable practices related to sweet talking and coaxing. Staff claimed that by coaxing and sweet-talking they avoided coercion. However, CKH observed that even though the strategies were for the patient’s benefit, sometimes staff’s actions could arguably approach coercion. For instance, in municipality 3, key informant Elise brings a patient to eat through a strategy which could be characterized as a threat:

When Elise returns, she is pleased with herself. She told the patient: ‘I will not leave until you have eaten’, and thereupon the patient started to eat (fieldnotes, municipality 3).

In this case, the patient’s autonomy was not preserved by sweet-talking and coaxing. Elise´s desire to provide what she thought was beneficent care was overridden by the fact that the patient did not want to eat.

During informal conversations and interviews, several informants said that they had ‘deceived’ patients into being placed in a nursing home. As described by key informant Silje from municipality 2:

When a patient is going to a nursing home, they have to deceive him. There is no point in telling him that he is going there because then he will not come with them. They can say they are going to a café or something nice. Then he will come with them. Silje knows this is really difficult and not okay, but she does not know any other way. ‘We cannot tie him up’ (fieldnotes, municipality 2).

In this situation, the patient did not want to move to a nursing home, but the staff used sweet-talking and coaxing to persuade him to do so. His autonomy and self-determination were not respected, but the patient’s life is no longer at risk. The informants considered this a necessity for the patient, as they could no longer provide him with the care, he needed at home.

#### Building trust over time

The second strategy to balance ethical challenges related to patient autonomy and provide beneficial and non-maleficent care was to build trust with the patient. This increased the likelihood that patients would accept care whilst the patient’s autonomy would be preserved.

The findings suggest that key informants build trust by taking time with the patient, that is, having conversations, going above and beyond their specified duties, telling personal stories, asking questions, being curious and listening to the patient, and not stressing.

During the focus group interview, informants in municipality 1 discuss the importance of knowing patients’ preferences – from small things such as what they like to eat on their bread to bigger issues such as how they want to die. They emphasise that all patients have a story and that they often do not know their story before going to the patient’s funeral, and that they wish they would have known more beforehand. Furthermore, the informants agreed that advanced care planning would benefit the patient and the care provided:

It would have been a good idea to have advanced care planning when home-based care starts to help them [the patients]. They are healthier than they will be later. And we learn their life story, how their life has been (Focus group interview, municipality 1). By getting to know the patient and building a relationship with them, home-based care workers were able to understand the patient’s preferences and values so that they could more easily identify how to adapt care to maintain the patient’s autonomy.

To be patient and show tolerance was another way of gaining the patients trust and thus avoid coercion. All informants explained that they often visited the patient many times over a long period before they were allowed to help. As Ida in municipality 3 expressed:

It often takes me two to three months to get in the right position to do something. It can be a long way to comb someone’s hair or change their blouse (…) It can take a whole year to get where you want to be (interview, municipality 3).

In many cases, CKH observed that patients and key informants talked about personal topics, knew the names of each other’s family members, knew what they had done over the weekend, and so on. The following description is a typical trust-building activity. Ida and CKH visited the patient Andrea, a woman living alone. After the necessary care was done, they sat with her on the sofa and talked:

We sit together with Andrea for a long time. We sit on the sofa; Ida has her leg curled up [on the sofa]. Andrea tells us about her life, that she has lost two children and that she loves the one she still has. We sit there for a long time and when we are outside, Ida says that we have used up twice the time, but that’s the way it is (fieldnotes, municipality 3).

By investing time and building a personal connection with the patient, staff were able to provide care because the patient trusted them and valued their opinion. For example, even if the patient initially refused food or help with personal hygiene, they eventually accepted it because they trusted that the staff had their best interests at heart; thus, the patient autonomy is preserved and the staff were able to give beneficial care.

## Discussion

The findings indicate that home-based care workers faced important ethical challenges related to patient autonomy and that they had developed strategies to deliver care while respecting patient autonomy.

### Balancing ethical principles

Ethics problems in clinical work often involve a conflict between the principle of autonomy and other ethical principles, not least the principle of beneficence. According to Beauchamp and Childress,^8^ the principle of beneficence ‘refers to a statement of a general moral obligation to act for the benefit of others’ (p. 218). This means that beneficence is not only about preventing harm but also about benefiting the patients and promoting their well-being.^43^ From the results, we can see that informants struggled to find a balance between providing beneficial and professionally sound care while preserving patients’ self-determination. When patients in home-based care reject the care proposed to them, staff had to decide which of the ethical principles weighed most heavily and sometimes find a middle ground. This is consistent with previous research showing that safety and harm prevention are essential moral concerns in home-based care.^9,15,16,18,19^

Preserving the patient’s autonomy can be particularly important in home-based care, as staff work in the patient’s home, amidst the patient’s personal belongings. The patient’s values and beliefs may also be more pronounced at home than in an institution. Thus, staff must take these preferences into account. Our findings indicate that home-based care workers give a large weight to autonomy, and one may question if the pendulum from paternalism to self-determination has swung too far in the direction of the latter.^8^ Paternalism can be divided into two types: hard and soft paternalism. Hard paternalism means intervening to prevent harm or benefit someone, even if that person’s choices are informed, voluntary, and autonomous. Soft paternalism, on the other hand, means intervening in the life of a person who is not able to exercise autonomy, on the basis of the principles of beneficence and non-maleficence.^8^ In some situations, informants seem to exercise soft paternalism vis-a-vis their patients. For example, Elliot, who was content with his situation and did not want a cleaner flat or other improvements suggested by the staff in home-based care. The concept of ‘ageing in place’ is a consequence of the shift in resources but also of the ‘grey wave’ societies are experiencing. However, many homes are not ‘aging ready’,^44^ that is, the home is not suitable for the infirm elderly, for example, with low or no thresholds, bedroom and bathroom on the first floor – as we have seen illustrated in the results. In many cases, patients’ homes are not only not ‘age appropriate’ but can also be directly dangerous. It was difficult to ensure the safety of the patients while maintaining their independence and autonomy. Previous research on home-based care emphasises the need for staff to understand and take into account patients’ life choices.^2^ The balance among the integrity of the individual, their independence, and the home environment is crucial for safety at home.^45,46^ Our findings suggest that the patient’s desire to live at home for as long as possible could pose some risk to the patient’s life and health. As Ekstedt et al.^47^ stated, the goal of home-based care is not ‘absolute safety’ but to consider the best interests of each patient. This meant considering the benefits of independent ageing at home while weighing the risks.

### The scale of influence

The informants in this study often exert influence on patients through sweet-talking and coaxing their patients over time, thus gradually ‘pushing’ them to accept the staff’s preferred course of action.

**Figure 1.**
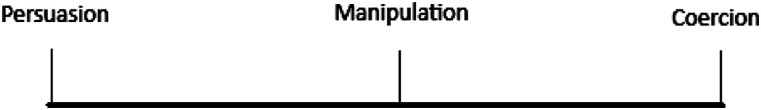
Influence.

Beauchamp and Childress distinguish between three categories of influence: coercion, manipulation, and persuasion. Coercion occurs when a person intentionally uses a credible and serious threat of harm or force to control another person.^8^ Manipulation is to get people to do what the manipulator wants them to do but not to coerce or persuade them, for example, to lie, to make people believe they have an autonomous choice, or to withhold information.^8^ Persuasion is when a person has been convinced by another person’s beliefs.^8^ We can see these as different degrees on a scale, with persuasion on one side, manipulation in the middle, and coercion on the other (Figure 1).

**Figure 2.**
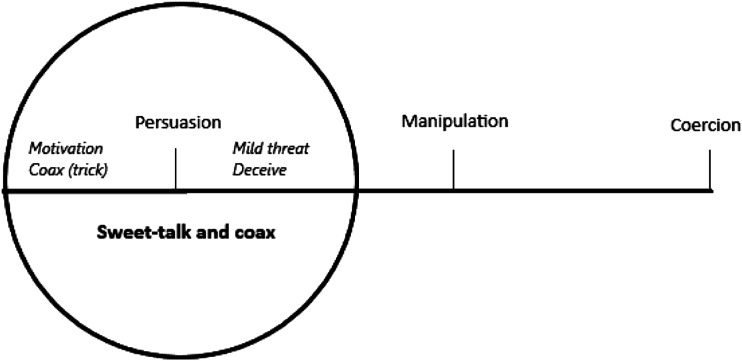
Sweet-talk and coax.

Influence can also include expressions of love, threats, education, lies, manipulative suggestions, and emotional appeals.^8^ It is instructive to attempt to relate the staff’s practices in the present study to Beauchamp and Childress’s classification. Considering the scale above, sweet-talking and coaxing should arguably be placed around ‘persuasion’. In many cases, the staff persuade the patient not primarily on the basis of staff’s own beliefs but based on the patient’s well-being, medical condition, and formal decision. Thus, this can be considered a milder form of influence than persuasion and could therefore fit in before persuasion. However, if we look at the situations where it was more complicated, where the boundaries were more blurred, as in the case of Elise telling a patient that she would not leave until he had eaten or enticing patients to move into nursing homes; sweet-talking and coaxing is closer to manipulation, but they do so to respect patient autonomy and for the benefit of the patient and not for themselves, thus it cannot be seen as fully manipulation (Figure 2

).

The scale above highlights the space in which the informants act. They mostly remain within the circle but can draw from a range of tactics which we argue are situated on both sides of ‘persuasion’.

Thus, we can say that ‘sweet-talking and coaxing’ include both positive and arguably more problematic actions. Since some behaviours and expressions tend towards the manipulative, we can ask whether these acts within sweet-talking and coaxing can go in the direction of informal coercion, as in the case of Elise who uses mild threat to get the patient to eat, or in the case of the staff deceiving patients to move into nursing homes. Hence, the line between sweet-talking, coaxing, and coercion can in some situations be blurred. In these cases, the informants navigate on a thin line and can be seen as ethically challenging situations.

In summary, staff sweet-talk and coax patients because they care about their well-being; they do it for the sake of beneficence. Even though there are positive and more questionable sides to sweet-talking and coaxing, it is a strategy for health workers to provide care and avoid risk and harm.

### Building trust requires time

It is essential to have a good relationship with a patient when using methods such as sweet-talking and coaxing or motivating.^48^ This can be seen as a therapeutic relationship where the roles of the nurse, the patient, and the family are established.^18^ They use trust-building not only to give the patient the appropriate care but also to make the patient feel safe and respected. However, it can be time consuming, depending on who is involved. Brodtkorb et al.^49^ investigated how caregivers and patients deal with the organisational system in home-based care. They found that rigid organisation made it difficult for staff to deviate from the patient’s formal allocation of care and attend to their actual needs. In home-based care, staff values and patient care are under constant pressure regarding efficiency and productivity.^50^ A key component in the healthcare sector is the commissioning model, where healthcare is defined by a formal decision rather than by the judgement of the one who performs care.^51^ This might be in tension with the moral obligation of the healthcare worker, as nursing is not a commodity or a service but a moral obligation springing from the patient’s needs.^52^

### A law not suited for the context?

Previous research on ethical challenges and coercion in home-based care shows that staff rarely use coercion and understand the concept differently.^22,25,26,49,53^ When coercion is used, the most common reasons are to preserve patient safety and to curtail aggressive behaviour.^23^

The key informants in our study were put in a difficult position when patients refused care. In such cases, informants claimed they did not use coercion because they were not allowed to. However, it seemed that the informants did not have a clear and settled understanding of the legislation. The Patient and User Rights Act^54^ does indeed allow health professionals to use coercion if a formal decision is made, often in cooperation with a physician, that is, the GP in home-based care. Gjellestad et al.^53^ found that in practice, the responsibility for deciding on coercive treatment in home-based care often lies with the patient’s GP. However, in home-based care the GP does not always know the patient or the situation. This might suggest that it is better for home-based care workers themselves to be responsible for making formal decisions about coercion.

Health professionals are responsible for keeping up to date with legislations.^55^ However, the law might be difficult to interpret and apply in the complicated context of home-based care. In addition, home-based care workers may be afraid of what it will mean for their relationship with the patient if they make a decision on coercion. As we saw in the findings, many of the informants were afraid of violating the patient’s trust. Coercing a patient could damage this trust and the long-built relationship.

### The need for clinical ethics support services

Arguably, home-based care staff would benefit from access to clinical ethics support services (CESS).^56,57^ CESS provide aid in formulating and discussing moral challenges, sometimes with a view to suggesting and justifying best courses of action in resolving the challenges. In Norwegian healthcare, ethics reflection groups (consisting of staff at a department) and clinical ethics committees (for the municipality’s entire health and care sector) are the most common types of CESS.^58,59^

In our view, CESS might be beneficial for home-based care in light of the challenges identified in the study. CESS might provide knowledge of legislation and ethical principles concerning coercion; a moral vocabulary; and reflection on the different kinds of influence and persuasion commonly practiced by staff.

## Limitations

The fieldwork began in September 2020 and ended in November 2021 and was impacted by the pandemic. There was a 2-month delay due to lockdown before the fieldwork in municipality 2 could commence. Other impacts included the need to use face masks, and limited meetings and informal contact with staff during some phases of the fieldwork.

CKH was aware that her role as a researcher could determine the data. She did what she could to act humble and curious so that her informants felt comfortable in her presence. However, key informants may have acted more cautiously around her, especially at the beginning of the fieldwork. Also patients may have displayed more gratitude and positivity with her presence than they otherwise would have. In addition, CKH is a young woman and mother, which could further influence the data obtained, as her informants were also women, many of them in the same situation. This role gained her the key informants trust as it was easy to find common ground.

The managers selected the employees who became key informants; conceivably, they might have selected ‘the best of the best’ in home-based care.

## Conclusion

Our findings show that home-based care staff experience ethical challenges when balancing patient autonomy and practicing beneficent care. To deal with these challenges, they employ strategies to respect patient autonomy while providing appropriate care to patients. We need to understand the ethical challenges that arise in home-based care as the population ages and the focus is on living at home as long as possible. Furthermore, it is difficult for home-based care staff to apply the law and ethical principles in their everyday work. Thus, it is important to do more research on this topic and by doing so we can develop ethical support for home-based care staff.
